# Model of Neuromorphic Odorant-Recognition Network

**DOI:** 10.3390/biomimetics8030277

**Published:** 2023-06-28

**Authors:** Sergey V. Stasenko, Alexey N. Mikhaylov, Victor B. Kazantsev

**Affiliations:** 1 Laboratory of Neurobiomorphic Technologies, Moscow Institute of Physics and Technology, 117303 Moscow, Russia; stasenko@neuro.nnov.ru (S.V.S.); kazantsev@neuro.nnov.ru (V.B.K.); 2 Laboratory of Advanced Methods for High-Dimensional Data Analysis, Lobachevsky State University of Nizhny Novgorod, 603022 Nizhny Novgorod, Russia; 3 Laboratory of Memristor Nanoelectronics, Lobachevsky State University of Nizhny Novgorod, 603022 Nizhny Novgorod, Russia

**Keywords:** spiking neural network, memristive synapse, neuron, olfactory analyzer

## Abstract

We propose a new model for a neuromorphic olfactory analyzer based on memristive synapses. The model comprises a layer of receptive neurons that perceive various odors and a layer of “decoder” neurons that recognize these odors. It is demonstrated that connecting these layers with memristive synapses enables the training of the “decoder” layer to recognize two types of odorants of varying concentrations. In the absence of such synapses, the layer of “decoder” neurons does not exhibit specificity in recognizing odorants. The recognition of the ’odorant’ occurs through the neural activity of a group of decoder neurons that have acquired specificity for the odorant in the learning process. The proposed phenomenological model showcases the potential use of a memristive synapse in practical odorant recognition applications.

## 1. Introduction

Volatile molecules, when inhaled through the nose, interact with molecular structures in the olfactory system of vertebrates, as described by Menini et al. [1]. The olfactory epithelium, situated within the nasal cavity, interacts with odor molecules, while olfactory sensory neurons serve as receptors, transmitting information about the binding processes between molecules to the brain through electrical signals. The process of odor perception entails the conversion of chemical interactions between olfactory receptors and volatile molecules into electrical signals, which convey information about the external world to the brain [1]. This information is relayed to the secondary neurons of the olfactory bulb (OB) and subsequently projected into the olfactory cortex and other regions of the brain, where patterns of information related to odors are encoded. The recognition of the specific molecular characteristics associated with each odor molecule involves the collaboration of various receptors, and the resulting pattern determines the perception of smell.

The biological olfactory system is renowned for its remarkable sensitivity and accuracy. The sense of smell possesses the remarkable ability to detect and differentiate tens of thousands of low-molecular-weight organic compounds. It can also recall memories associated with diverse organic polymers, including alcohols, esters, carboxylic acids, ketones, sulfides, nitriles, thiols, immigration, halides, and ethers [2]. As a result, the intricate mechanism of smell has garnered significant interest in the field of cognitive engineering in recent years.

Across different organisms, the sense of smell has developed in a similar way. Odor discrimination occurs when odor molecules interact with receptor residues [3]. This interaction bears similarities to the binding process observed between antigens and antibodies in the immune system, as well as neurotransmitters and receptors in the nervous system [4]. Odor molecules, referred to as odogens, function as epitopes in this context. When a receptor binds with an odor molecule, the chemical energy of the interaction is transformed into a nerve signal, which is subsequently transmitted through the olfactory bulb (OB). The OB processes odor information and transmits it to other brain regions. The brain recognizes each odor molecule based on a unique combination code formed by the signals from multiple olfactory receptors [5]. Even a slight structural difference in the odor molecule can result in a different odor perception due to a distinct combination code. While the exact functional role of the OB’s signaling process remains uncertain [6], it plays a critical role in olfactory processing.

For approximately 40 years, scientists have been captivated by artificial olfactory systems. In 1982, Persaud and Dodd published a significant paper demonstrating the capability of four chemical sensors with overlapping selection patterns to distinguish different odors [7]. The pattern of signal combination in each receptor ensemble plays a vital role in odor classification, identification, and recognition [8]. Since then, the majority of olfactory sensor technologies have employed sensor arrays designed for the purpose of odor classification. An electronic nose (e-nose), which mimics the olfactory recognition system found in humans by utilizing an array of electronic sensors, has been developed and represents a notable example of an artificial olfactory system [9].

An electronic nose has been developed, which integrates an array of gas sensors with overlapping selectivities to capture the signatures of encountered odors. The system translates variations in the characteristics of each sensor into electrical signals and employs gas-identification algorithms based on these signatures to differentiate between different odors. This approach is particularly useful for odors that may demonstrate significant variability within a class but limited separation between classes.

Since its development, the electronic nose has found applications in various sectors, including medicine, the environment, agriculture, manufacturing, and the military. In the medical field, it has been utilized for detecting lung cancer, Alzheimer’s disease, and Parkinson’s disease. In the environmental sector, it is employed for monitoring air and water quality. In agriculture, it aids in inspecting food quality and identifying pre- and post-harvest diseases. The manufacturing sector benefits from its use in ensuring product uniformity and preventing workplace health hazards. Lastly, in the military, it is employed for detecting explosives and chemical weapons [10,11]. Research on electronic nose (EN) applications has significantly grown, especially in the food industry [12,13,14,15]. ENs have the ability to detect food contamination, assess quality, control shelf-life and spoilage, and identify hazardous chemicals or bacteria in food and beverages. The presence of microorganisms, enzymes, and oxidation in food production and storage can lead to deterioration and the growth of harmful bacteria or nutrient loss. Luckily, ENs can detect the chemical gases released by spoiled food, addressing important food safety concerns. Air pollution is a serious health risk and diminishes quality of life in developed and developing countries [16]. Reducing unpleasant odors emitted from sewers and wastewater treatment plants is a crucial objective [17]. To tackle these challenges, the detection of toxic gases responsible for pollution is essential. ENs have emerged as a promising technology to address these issues, as evidenced by numerous studies [12]. ENs are widely utilized in security applications due to their selectivity, portability, wide linear range, low cost, and compactness [12]. They have gained significant attention for national security purposes, particularly in detecting explosive compounds [18]. ENs are also employed in monitoring and securing confined spaces, as demonstrated by an experiment using metal-oxide sensors, along with oxygen, temperature, and humidity sensors, to analyze atmospheric air samples successfully [19].

Microelectronics could greatly benefit from memristive nanodevices, which provide compact, multi-level, and non-volatile memory functionality [20]. However, their inherent variability [21,22] can pose challenges in fully harnessing their potential, necessitating more robust architectures. Spiking neural networks, inspired by the brain’s variable neurons and synapses [23], offer a promising solution with high efficiency. The neuromorphic community has been exploring this concept using CMOS circuits to model spiking neurons and synapses, but limitations arise in terms of the number of implementable synapses [24,25]. Memristive nanodevices, such as resistive RAMs, memristors [20], or adaptive transistors [21], can provide the compact synapses required for more advanced neuromorphic circuits. Recent studies have demonstrated that these devices can replicate biological synapses’ learning rules, such as spike timing-dependent plasticity (STDP), and even more complex plasticity rules crucial for brain-inspired learning [26,27,28,29,30]. By combining memristive synapses with CMOS neurons (or potentially memristor-based neurons in the future [31]), significant advancements in computing and highly efficient cognitive tasks could be achieved.

In this article, we propose a new model of a neuromorphic olfactory analyzer based on a memristive synapse. The model consists of two layers of neurons connected by memristive synapses, which make it possible to train a layer of “decoders” to recognize two types of odorants of different concentrations. In the absence of such synapses, the layer of “decoder” neurons does not show specificity in the recognition of odorous substances. The recognition of odorants is achieved through the neural activity of a group of decoder neurons that acquire specificity for the odorant during the learning process. The proposed phenomenological model is based on neuronal synchronization observed in experimental studies on odor recognition [32]. Both experimental and modeling studies have demonstrated that correlated inputs enhance neuronal firing [33], with synaptic plasticity mechanisms favoring correlated synaptic inputs [34].

## 2. The Model

The proposed olfactory model consists of two layers comprising pre- and postsynaptic neurons. The presynaptic neurons represent a receptive field composed of olfactory neurons in contact with an odorant, while the postsynaptic neurons are detector neurons responsible for recognizing a specific odorant and its concentration.

### 2.1. Odorant Concentration

The fluctuations in the concentration of an odorant are modeled using a process known as the half-wave rectified Ornstein–Uhlenbeck process [35]:

(1) $$\tau \frac{dx}{dt}=-x+\sqrt{2\tau }\xi \left(t\right),$$

The time constant τ=75ms corresponds to the odorant concentration, which is proportional to the half-wave rectified Ornstein–Uhlenbeck process ${\left[x\right]}^{+}=\max\left(x,0\right)$. The olfactory receptor system consists of N=5000 neurons, each with a specific affinity and global sensitivity for different types of odorants. An odorant can be represented as an N-dimensional vector of binding coefficients $b_{i}$, which are the product of the affinity $a_{i}$ and sensitivity $s_{i}$ of each receptor neuron. The binding coefficients are randomly generated with a logarithmic distribution between $10^{-3}$ and $10^{3}$ to mathematically define the odorant.

When an odorant binds to the receptors of an olfactory neuron, a membrane current is generated. The Hill function is used to simulate the process of odorant binding to receptors and the formation of a response in the form of a current in the olfactory system [35,36]. The parameters of the function were obtained from previous research [37]. This current can be approximated by a Hill function that depends on the concentration of the odorant:

(2) $$I=I_{\max}\frac{c^{n}}{c^{n}+K_{\frac{1}{2}}^{n}}=I_{\max}{\left(1+{\left(K_{\frac{1}{2}}/c\right)}^{n}\right)}^{-1},$$

The equation represents a Hill function that depends on the time-varying concentration of the odorant, denoted by *c*. The Hill coefficient is denoted by *n*, which is equal to 3 and determines the slope of the curve. The maximum velocity, denoted by $I_{max}$, is calculated to be 40 Hz. The semi-activation concentration, denoted by $K_{1/2}$, is the reciprocal of the binding coefficient, denoted by $K_{1/2}=1/b_{i}$. The concentration fluctuates randomly over time, and its dynamics are described by the equation $c\left(t\right)=c_{0}\;\cdot \;x\left(t\right)$.

### 2.2. Olfactory Neuron

**Figure 1:**
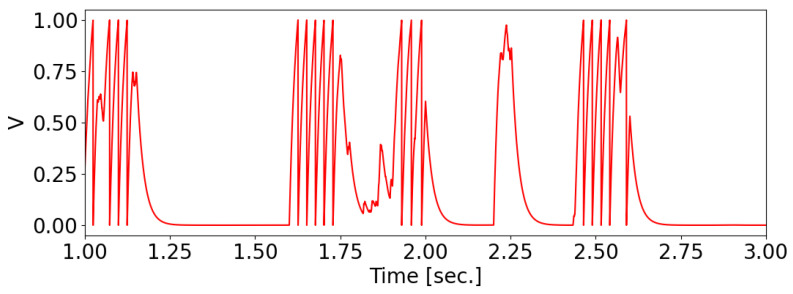
The membrane potential of the olfactory neuron in the receptive field changes over time.

When an odorant binds to the receptors of a receptive neuron, it generates a current across the neuron’s membrane, causing a change in its potential and the production of a series of spikes on the membrane. The membrane potential of the olfactory neuron is characterized by the “integrate-and-fire” model [38].

(3) $$\tau \frac{dv_{i}}{dt}=-v_{i}+I_{i}\left(t\right),$$

where τ=20 ms is the membrane relaxation time constant and $I_{i}\left(t\right)$ is the membrane current. A spike occurs when $v_{i}=1$, after which the membrane potential resets to 0 (see Figure 1).

### 2.3. Neuron Detector

The “integrate-and-fire with noise” model was employed to simulate the behavior of the postsynaptic neuron, also referred to as the detector neuron, as follows:

(4) $$\begin{array}{c} \tau \frac{dv_{j}}{dt}=-v_{j}+\nu_{i}+I_{syn_{j}}\\ \tau \frac{d\nu_{j}}{dt}=-\nu_{j}+\sigma \sqrt{2\tau }\phi \left(t\right) \end{array}$$

where τ=5ms is the membrane relaxation time constant. The filtered noise input $\nu_{j}\left(t\right)$ had a standard deviation of σ=0.2, where ϕ(t) is white noise. As a result, the standard deviation of the resulting membrane potential $v_{j}$ was approximately $\sigma_{v_{j}}=\sigma /\sqrt{2}\approx 0.14$.

**Figure 2:**
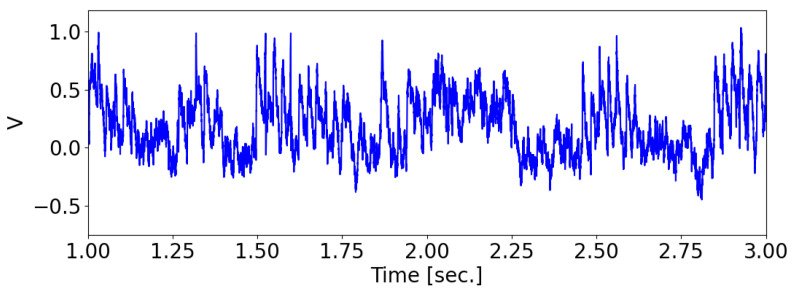
The membrane potential of the neuron-detector changes over time.

Whenever a presynaptic neuron spikes, the membrane potential $v_{j}$ of the postsynaptic neuron is incremented by 1/N, where *N* is the total number of presynaptic neurons. A spike is registered when the membrane potential $v_{j}$ reaches 1. The factor of 1/N in scaling guarantees that the postsynaptic neuron will have a firing probability of 1/2 when the signals from presynaptic neurons are synchronized. After a spike, the membrane potential is reset to 0 (see Figure 2).

The current flowing through the synapse of the postsynaptic neuron, denoted as $I_{syn_{j}}$, is the sum of all synaptic currents generated by the presynaptic neurons *M* connected to the postsynaptic neuron.

(5) $$I_{syn_{j}}=\sum_{i=1}^{M}w_{i,j}.$$

### 2.4. Memristive Synapse

In our model, synapses between pre- and postsynaptic neurons are represented as resistors that transmit spikes with varying conductance. The conductance in the model corresponds to the synaptic weights, denoted as *w*. Furthermore, the model incorporates the adaptation of conductance based on neuron activity, which is essential for training the model.

**Figure 3:**
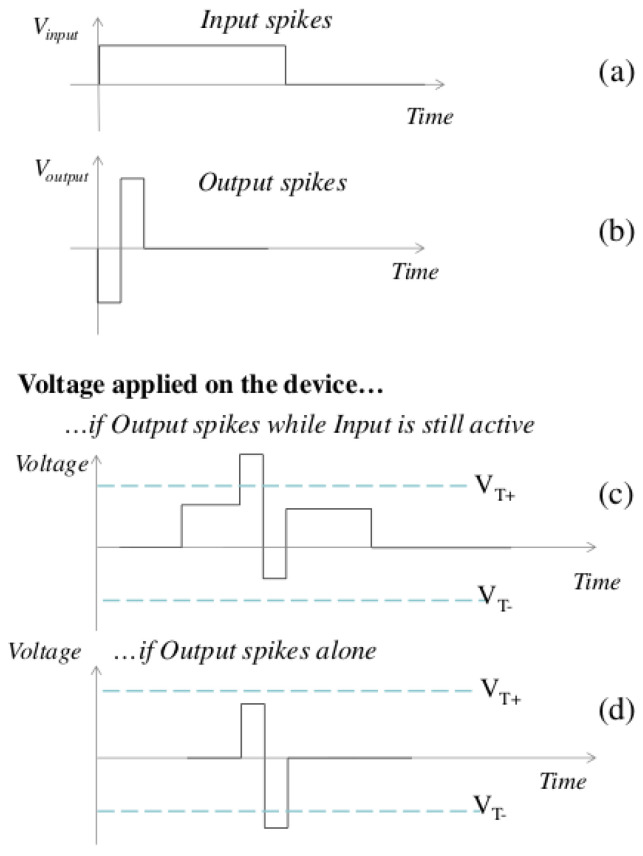
The simplified STDP training involves voltage pulse versus time as follows: (**a**) A spike generated on the presynaptic neuron leads to transmission of the input pulse to the memristor. (**b**) When the postsynaptic neuron produces a spike, the output pulse is transmitted to the memristor. The conductance of the memristor increases or decreases when the voltage applied to the device (the difference between the voltages applied at the two ends) (**c**) or (**d**) reaches $V_{T+}$ or $V_{T-}$, respectively [39].

In experimental settings, memristive devices are programmed to increase their conductance when a positive voltage pulse is applied above the threshold $V_{T+}$, while they decrease their conductance when a voltage pulse is applied below the negative threshold $V_{T-}$ [27]. Previous research has demonstrated that memristive devices can implement spike-timing-dependent plasticity (STDP), which is a learning rule observed in biological systems [27,29]. However, the proposed model utilizes a simpler learning scheme based on pattern extraction, as illustrated in Figure 3.

**Figure 4:**
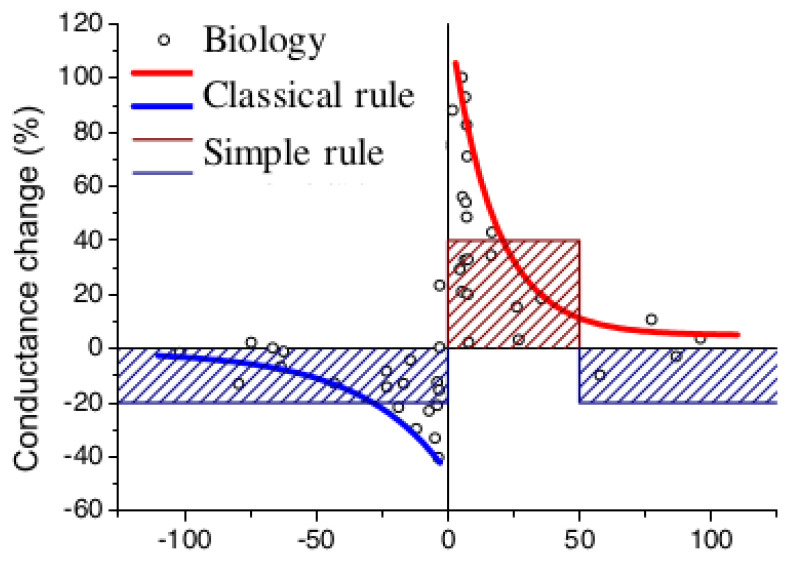
A simplified STDP rule compared to the standard biological STDP rule [39].

When a presynaptic neuron generates a spike, a prolonged voltage pulse is applied to the synapses, enabling the postsynaptic neuron to integrate the resulting current (Figure 3a). If multiple synapses associated with the same postsynaptic neuron are active simultaneously, their currents are summed. When a spike is generated on a postsynaptic neuron, an impulse is transmitted to the synapses, resulting in the formation of a positive or negative bias (Figure 4 compares the simplified STDP rule with the classic biological STDP).

The evolution of the memristor’s conductance follows the Querlioz model [40], which is based on experimental findings on memristors [27]. The increase in conductance can be mathematically represented by the following equation:

(6) $$\begin{array}{c} \delta w_{p}=\alpha_{p}e^{-\beta_{p}\frac{w\left(i,j\right)-w_{min}}{w_{max}-w_{min}}} \end{array}$$

While the increase in conductance of the memristor can be mathematically formalized using the previous equation, the decrease in conductance can be expressed as follows:

(7) $$\begin{array}{c} \delta w_{m}=\alpha_{m}e^{-\beta_{m}\frac{w_{max}-w\left(i,j\right)}{w_{max}-w_{min}}} \end{array}$$

The values of the parameters $\alpha_{p},\beta_{p},\alpha_{m},\beta_{m}$ are highly dependent on the specific input and output voltage impulses that are used. Furthermore, the minimum and maximum conductances $w_{min}$ and $w_{max}$, as well as these parameters, may vary between different physical devices.

### 2.5. Neural Network

The neural network consists of 5000 olfactory neurons (presynaptic) in the receptive field, to which odorants are applied, and 30 detector neurons (postsynaptic) that respond to odorants and their concentrations. The neurons are interconnected using the “all-to-all” connection type, and the probability of connection is equal to 1.

**Figure 5:**
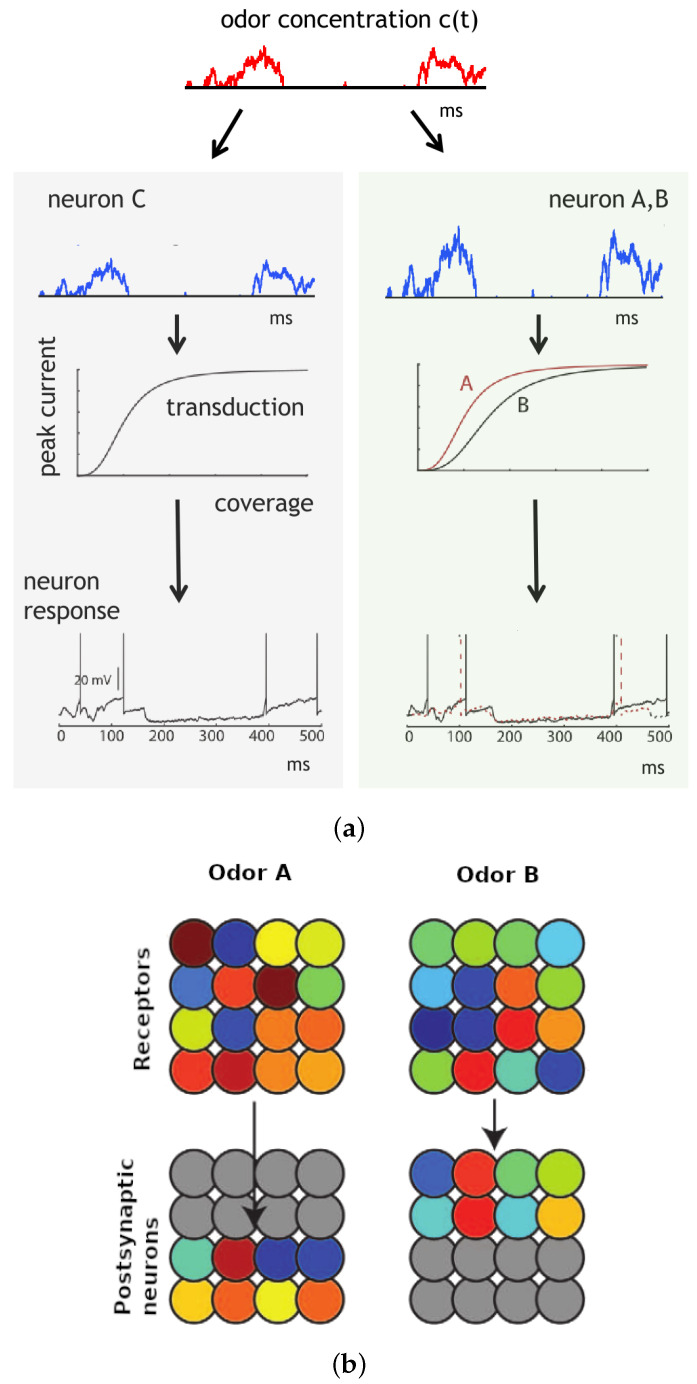
Schematic view of odorant recognition in the neuromorphic network model. (**a**) At the top of the figure, an odor is presented at varying concentrations. The amount of receptor coverage depends on both the concentration and receptor type, with type 2 green area) receptors being more sensitive to the odor than type 1 (gray area) receptors. The transduction current, depicted in the middle, is determined by the peak transduction current, which is a function of receptor coverage that varies among different neurons due to their varying half-activation coverages (global sensitivity). Neurons fire in synchrony to an odor when the product of odor affinity and global sensitivity match. Neurons B and C exhibit this synchrony (black traces), while neurons A and C do not (dashed red trace). (**b**) The top part of the figure shows that various odors result in distinct synchrony partitions. Receptors with the same color represent synchronized signals. The bottom part of the figure demonstrates that each odor corresponds to a specific set of postsynaptic neurons, where the inputs to each neuron originate from the same synchrony group. In each column, each postsynaptic neuron with a particular color receives synapses from all receptors with the same color [35].

In Figure 5, our olfactory model exhibits odor-specific synchrony [35]. The concentration of the odor, denoted as c(t), undergoes random fluctuations caused by turbulences, while the coverage of receptors is influenced by both the concentration and the specific receptor type. Receptor type 2 (green area) exhibits higher sensitivity to the odor compared to receptor type 1 (gray area) (see Figure 5a). Subsequently, the odor is transformed into an electric current that generates spikes. The transduction current is represented by a Hill function of receptor coverage, with the Hill coefficient reflecting the steepness of the curve [41]. Neurons exhibit synchronous firing when they receive identical input, which occurs when they possess the same affinity and global sensitivity. Conversely, neurons with different affinities and global sensitivities do not fire synchronously. Importantly, the synchrony phenomenon is not dependent on odor concentration.

Figure 5b illustrates the response of a population of olfactory neurons to different odors. Every odor is depicted by a randomized vector of affinities, and the odor concentration is simulated using a noise signal that undergoes a half-wave rectification and low-pass filtering. Both the receptors and postsynaptic neurons follow noisy integrate-and-fire models with randomized global sensitivity. Each odor elicits a distinct pattern of synchrony in the receptors, which can be interpreted by the postsynaptic neurons. Upon the presentation of a particular odor, postsynaptic neurons connected to the corresponding synchrony pattern become active, while neurons tuned to other odors remain inactive. However, due to their wide-ranging tuning, the majority of receptor neurons respond to both odors.

### 2.6. Numerical Simulation and Data Analysis Methods

For numerical calculations, the Euler method and the Euler–Maruyama method for stochastic differential equations were used with an integration step of 0.01. All calculations were carried out using software written in Python using libraries for scientific computing and data analysis [42,43].

## 3. Results

In order to investigate the impact of a synapse, in the form of a memristor, on the connection between olfactory neurons in the receptive field and detector neurons that identify the presented odorant, we examined two scenarios: a simple synapse with a constant synaptic connection, and a memristive synapse that adaptively adjusts the strength of the connection between neurons.

During training, the neural network was presented with two types of odorants every 200 ms, randomly, for a period of 100 s. The final weights were saved after the 100-s training session and used during testing.

**Figure 6:**
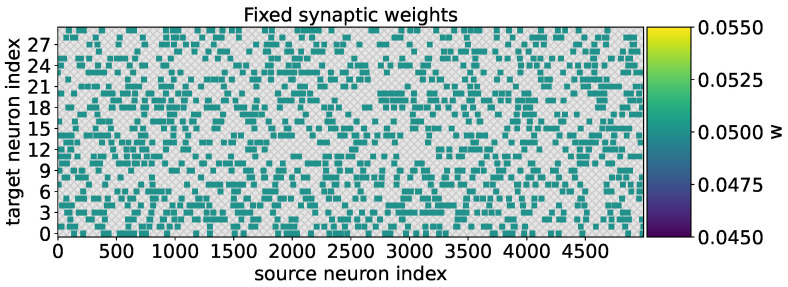
The matrix of neural network weights between presynaptic neurons (source neuron index) and postsynaptic neurons (target neuron index). The colors represent the weight values between presynaptic neurons (source neuron index) and postsynaptic neurons (target neuron index).

In the case when the neural network was trained to recognize the two types of odorants within 100 s without adaptive weight adjustment, the weight values remained fixed for all the formed synapses (Figure 6).

The procedure for testing the neural network to recognize the presented odorant was carried out as follows:

1. The weights of the neural network were set according to the values obtained during training.
2. Odorant A was supplied for 20 s, followed by odorant B for 20 s.
3. The concentration of the odorant was increased by one every second.

**Figure 7:**
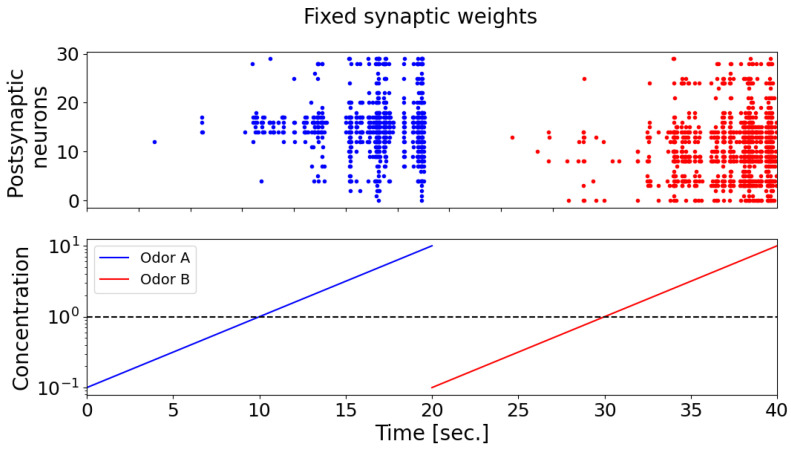
The raster plot of spike activity of postsynaptic neurons (top graph) during the presentation of odorants A (blue) and B (red) with increasing concentration (from 0.1 to 10, where 1 is the concentration of the odorant during the learning phase). In the case of fixed synaptic connections, postsynaptic neurons form a similar activation pattern (blue and red dots on the raster plot of spike activity (upper panel)), capturing almost all neurons with an increase in the concentration of odorant A (blue curve) and odorant B (red curve), and showing minimal neuronal activation at low concentrations of odorants (bottom panel).

During the testing of odorants, it was found that the postsynaptic neurons (detector neurons) exhibited weak selectivity with respect to the supplied odorants (Figure 7). It can also be seen that, when the concentration of odorants is less than 1, postsynaptic neurons almost do not respond. In Figure 7, the raster diagram of neural activity in response to the presentation of odorant A is marked in blue, and odorant B is marked in red. When synaptic connections are fixed, postsynaptic neurons exhibit a comparable pattern of activation, as depicted by the blue and red dots on the spike activity raster plot (the upper panel). This pattern captures the majority of neurons, demonstrating increased activity in response to higher concentrations of odorant A (the blue curve) and odorant B (the red curve), as well as exhibiting minimal neuronal activation at lower odorant concentrations (bottom panel).

**Figure 8:**
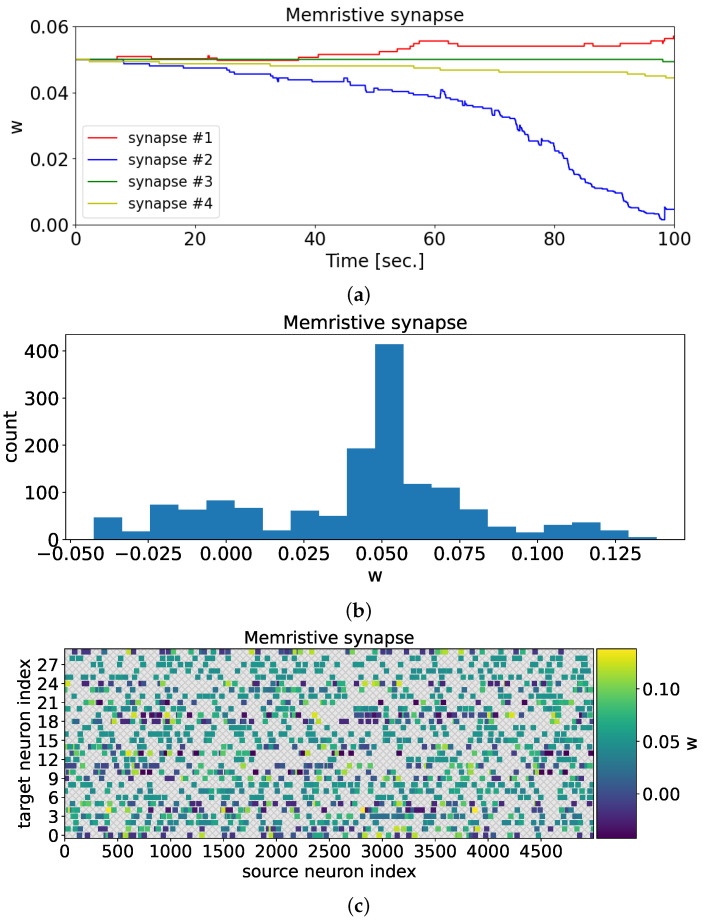
Characteristics of synaptic weights in the case of memristive plasticity. (**a**) The change of synaptic weights for synapses 1, 2, 3, and 4 during neural network training. (**b**) The distribution of synaptic weights after training. (**c**) The matrix of neural network weight values between presynaptic neurons (source neuron index) and postsynaptic neurons (target neuron index). The colors represent the weight values between presynaptic neurons (source neuron index) and postsynaptic neurons (target neuron index). Compared to Figure 6, synaptic weights assume different values. Memristive plasticity results in a modification of the synaptic weight values, enabling the spiking neural network to exhibit selectivity towards different odorants.

In the case of a memristive synapse, after training the neural network, one can observe the adaptation of synaptic weights to the presented odorants (Figure 8). Figure 8a illustrates the changes in synaptic weights during the 100-s training period. In contrast to Figure 6, the synaptic weights in the current scenario adopt distinct values. Through memristive plasticity, the synaptic weight values undergo modifications, allowing the spiking neural network to demonstrate selectivity towards various odorants.

Once the training process was complete, the final weights were determined and remained fixed for the subsequent testing of the model.

**Figure 9:**
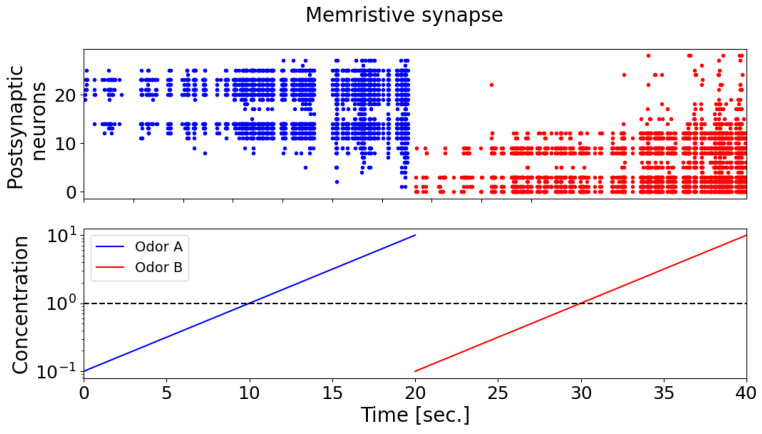
The raster plot of spike activity of postsynaptic neurons (upper graph) during presentation of odorants A (blue) and B (red) with increasing concentration (from 0.1 to 10, where 1 is the concentration of the odorant in the learning phase). Postsynaptic neurons, in the case of memristive synaptic connections upon the presentation of odorants A (blue curve) and B (red curve) exhibit a selective response characterized by contrasting activation patterns (blue and red dots on the raster plot of spike activity (upper panel)). As the concentration of odorant A increases (blue curve (lower panel)), neurons with indices of 11 and higher are predominantly activated, gradually increasing the number of responding neurons and forming their unique activation pattern (blue dots on the raster plot of spike activity (upper panel)). Subsequently, the presentation of odorant B (red curve (lower panel)) results in an opposite activation pattern (red dots on the raster plot of spike activity (upper panel)), with predominant activation observed in neurons with indices up to 10.

The results show that, with the memristive synapse, the neural network was able to successfully recognize the two presented odorants (as shown in Figure 9). Upon the introduction of odorants A (the blue curve) and B (the red curve) in the case of memristive synaptic connections, postsynaptic neurons display a selective response characterized by distinct activation patterns (the blue and red dots on the spike activity raster plot, the upper panel). With an increasing concentration of odorant A (the blue curve, the lower panel), neurons indexed from 11 onwards exhibit predominant activation, progressively increasing the number of responsive neurons and forming a unique activation pattern (the blue dots on the spike activity raster plot, the upper panel). Subsequently, the presentation of odorant B (the red curve, the lower panel) elicits an opposing activation pattern (the red dots on the spike activity raster plot, the upper panel), with predominant activation observed in neurons indexed up to 10. Notably, as the concentration of odorants increased, the number of activated postsynaptic neurons also increased.

## 4. Discussion

The electronic nose mimics the biological olfactory system using an array of gas sensors and odor-classification algorithms to identify odor fingerprints. However, challenges remain in making it comparable to its biological counterpart, such as limited sensor numbers, high power consumption, and inflexible operating conditions. The major challenge is to find a hardware-friendly, fast gas-identification algorithm with high classification performance. The electronic nose has applications in flavor, fragrance, food and beverage, packaging, pharmaceutical, cosmetic, perfumes, and olfactive nuisance monitoring. It can track fluctuations and trends in real-time, leading to proactive odor management, and existing commercial systems can be programmed with active alerts based on set points.

An electronic circuit with a memristor was used to model the adaptive behavior of unicellular organisms [44]. This circuit could have applications in pattern recognition. The DARPA SyNAPSE project and other researchers are developing neuromorphic architectures using memristive systems [45,46]. In 2013, Leon Chua highlighted the diverse range of phenomena and applications that memristors can mimic, including classic habituation and learning [47].

The active development of odor analysis systems and memristive devices, as well as their practical significance, have made their combination relevant. For example, in the work [48] to detect counterfeit beer in China, researchers designed an electronic nose system with 4 MOS gas sensors, a sample-delivery subsystem, and a software-based data acquisition subsystem. They used a memristor back-propagation neural network to classify the quality of the beer. In another work [49], an artificial olfactory system based on memristive devices was developed to classify four gases with ten concentrations. The system uses a reservoir computing system to extract spatiotemporal features, which are then processed by a classifier based on nonvolatile memristive devices. In the work [50], a novel approach for electronic nose odor recognition using a memristive convolutional neural network (CNN) was developed. The proposed network achieved a remarkable accuracy of 99.72% across six odor categories while utilizing only 56 weight parameters. By leveraging a simple network structure and an ultra-tiny memristor array, the system enhanced efficiency and recognition accuracy. Additionally, the approach significantly reduced calculation time and computing power requirements. An electronic nose (e-nose) based on memristive devices has emerged as a promising approach in the field of olfactory technology. Memristive devices, including memristors and memristive crossbar arrays, offer unique characteristics that can enhance the performance of electronic nose systems.

To implement our model in a practical system, it may be necessary to convert the studied odors into an electrical signal. One promising technology for odor coding is gas chromatography-olfactometry (GC-O) [51]. GC-O combines traditional gas chromatographic analysis with sensory detection to study complex mixtures of odorous compounds [52]. This technique involves a split-column GC system with a conventional detector (e.g., a flame-ionization detector or mass spectrometer) and a sniffing port. The sniffing port, made of glass or PTFE, is designed to fit the shape of the nose and delivers the eluate through a heated transfer line to prevent condensation. Humid air is added to the eluate to prevent the drying of the nasal mucous membrane during prolonged analysis [53,54]. The sensory responses are recorded in an olfactogram, enabling the simultaneous comparison of the chromatogram and olfactogram [53].

The essential difference in the model we propose is that we aim to strike the best compromise between the bioavailability of the system and its simplicity, allowing us to develop an electronic device based on it. Moreover, in contrast to the reported data obtained for formal neural classifiers using memristors as programmed synaptic coefficients, in our work, the self-learning of memristive synapses is implemented depending on the local activity of spiking neurons. The undisputed advantage of our work is the introduction of a phenomenological biophysical model of a spiking neural network with memristive synapses, which emulates recognition processes based on neuronal synchronization found in biological systems [32]. However, a drawback of the proposed model is its qualitative nature, focusing on reproducing the biophysical process of odor recognition in the brain (activation of neuron groups) rather than addressing quantitative parameters crucial in practical domains. Existing machine learning metrics are inapplicable to the proposed model, preventing direct comparison with current implementations of “electronic nose” systems utilizing artificial neural networks and machine learning for specific applications in medicine, nutrition, environmental monitoring, and security [12]. The results of our study, combined with the existing architectures of memristor-based spiking neural networks, can enable the implementation of brain-like and energy-efficient neural networks for processing multidimensional and analog information [55].

## 5. Conclusions

A neuromorphic olfactory analyzer model based on a memristive synapse is introduced. The model consists of two layers of neurons linked by memristive synapses, which enable the training of a layer of “decoders” to distinguish between two types of odors with different concentrations. Without such a synapse, the layer of “decoder” neurons lacks specificity in identifying odorous substances.

## Data Availability

The data that support the findings of this study are available from the corresponding author upon reasonable request.
